# Investigation into the interchangeability of generic formulations using immunosuppressants and a broad selection of medicines

**DOI:** 10.1007/s00228-015-1878-z

**Published:** 2015-06-12

**Authors:** Yang Yu, Steven Teerenstra, Cees Neef, David Burger, Marc Maliepaard

**Affiliations:** Department of Pharmacology and Toxicology, CARIM, Maastricht University Medical Centre, Maastricht, The Netherlands; Medicines Evaluation Board, Utrecht, The Netherlands; Department for Health Evidence, Section Biostatistics, Radboud University Nijmegen Medical Centre, Nijmegen, The Netherlands; Department of Pharmacology and Toxicology, CAPHRI, Maastricht University Medical Centre, Maastricht, The Netherlands; Department of Pharmacy, Radboud University Nijmegen Medical Centre, Nijmegen, The Netherlands

**Keywords:** Generic drugs, Interchangeability, Bioequivalence, Pharmacokinetics

## Abstract

**Purpose:**

To date, the interchangeability of generic drugs has only been investigated for a limited number of medicines. The objective of this study was to investigate generic-generic drug interchangeability in a large subset of generic formulations in order to cover a broad spectrum of drugs.

**Methods:**

Orally administered drugs for investigation in this study were selected using strict, predefined criteria, with the purpose to avoid bias. This selection procedure yielded atorvastatin, bicalutamide, naratriptan, olanzapine, perindopril, and venlafaxine. Further, ciclosporin, tacrolimus, and mycophenolate mofetil were investigated as test immunosuppressants. Adjusted indirect comparisons were conducted between generic drugs containing the same active substance, and the 90 % confidence interval (CI) for AUC and C_max_ was calculated.

**Results:**

In total, 120 bioequivalence studies were identified in the Dutch medicine regulatory agency’s database, allowing 292 indirect comparisons between generic drugs. The indirect comparison results indicated that in the vast majority of cases, i.e., 80.5 %, the 90 % CIs for both AUC_t_ and C_max_ fell within the bioequivalence criteria (in 90.1 and 87.0 % for AUC_t_ and C_max_, respectively). In 1 % of the 292 indirect comparison for AUC_t_ and 3 % for C_max_, a wider range of 75–133 % (or 80–125 %) was exceeded.

**Conclusions:**

Overall, our study suggests that exposure-related risks associated with the exchange of different generic drugs in clinical practice are not increased to a relevant extent compared to the situation in which a generic is exchanged with the innovator.

**Electronic supplementary material:**

The online version of this article (doi:10.1007/s00228-015-1878-z) contains supplementary material, which is available to authorized users.

## Introduction

A generic medicinal product is considered to be therapeutically equivalent to the innovator. To be registered, one or more so-called bioequivalence studies are required to demonstrate that the 90 % confidence interval (CI) for the generic/innovator ratios of the area under the drug concentration-time curve (AUC) and the maximum concentration (C_max_) is within the range of 80–125 % [1–4]. This acceptance range of 80–125 % can be widened based on a scaled approach for C_max_ up to 69.84–143.19 % for highly variable drugs or can be tightened to 90–111.11 % for narrow therapeutic index drugs (NTIDs).

At present, for most active pharmaceutical ingredients (APIs), multiple generic products have been approved in the Netherlands, and regular switches from one generic product to another are seen in clinical practice. From a regulator’s perspective, generic-generic drug switching is unlikely to impact treatment, as all generic formulations have been shown to be bioequivalent to the innovator product, and thus, the deviation of drug exposure between generic products should be limited [5]. Based on that, generic products are considered to be sufficiently comparable to each other. However, current regulation does not require bioequivalence studies between different generic formulations. In fact, in theory, a “drift” may appear upon generic-generic drug substitution, meaning that generic formulations that are bioequivalent to the innovator drug, respectively, may not be bioequivalent to each other [6]. This potential problem is due to the acceptance range for generic product registration (90 % CIs of AUC and C_max_ ratios within the 80–125 % range), which allows small variations in exposure between generic and innovator drugs. Thus, if one generic product has a higher and another has a lower exposure level than the innovator drug, the difference between the generic drugs will be reinforced, potentially leading to bio-inequivalence between them. In addition, the possibility of the occurrence of bio-inequivalence between generic drugs is also shown in theoretical Monte Carlo simulation studies [7, 8]. Therefore, investigation into this generic-generic drug comparability is warranted.

In light of the discussion about the drift upon generic drug substitutions, we have previously conducted indirect comparisons to evaluate generic-generic drug interchangeability using gabapentin and topiramate as test medications [9]. As a result, in general, bioequivalence between the different generic gabapentin and topiramate formulations was demonstrated. These interstudy comparison results were subsequently confirmed and validated by a clinical bioequivalence study using multiple generic formulations of gabapentin [10]. Furthermore, in the public domain, the comparability and safety of generic immunosuppressants, typically in transplantation medicines, are hot topics of discussion [11–17]. Therefore, this study is conducted to investigate the acceptability of generic-generic drug interchangeability for a broad spectrum of medicines as well as immunosuppressants based on the bioequivalence studies submitted for registration.

## Methods

### Drug selection

For this study, only orally administrated tablets or capsules with systemic action were considered relevant. The database at Dutch medicine regulatory agency was used for drug selection and data collection. To avoid a selection bias, a period of January 1 to May 9, 2008 was predefined to create a cohort, which contains all APIs that had at least one generic formulation (tablets or capsules) registered during this period, regardless of the type of registration procedure. Second, the identified APIs were ordered according to the registration date starting from January 1, 2008. In order to largely predict the interchangeability of currently marketed generic drugs, it was predefined to select the first six APIs from the initial cohort that had more generic drugs registered after January 1, 2008 (until June 8, 2010). In addition to this predefined selection, we also decided to investigate cyclosporine, tacrolimus, and mycophenolate mofetil as test immunosuppressants, triggered by the debate in the field on generic drugs from this class. For all selected APIs, bioequivalence studies in the registration files of generic formulations before January 1, 2012 were retrieved from the Dutch medicine regulatory agency’s database.

### Data analysis

Adjusted indirect comparisons between generic products containing the same API were conducted. This method has been well used in this kind of research, since it allows an estimation of the 90 % CI based on an interstudy comparison. The major limitation is that the uncertainty (i.e., standard error) of the indirect comparison is larger than the standard error of any of the studies under comparison, which is expected to be also larger than the standard error in case two generic drugs are directly compared in a study. It therefore leads to an overestimation of the differences between generic drugs (i.e., broader 90 % CIs than calculated in a direct comparison). Thus, the adjusted indirect comparison method is considered as a conservative approach and is expected to provide reliable results in the case that the adjusted indirect comparison indicates the 90 % CIs within the acceptance range of 80–125 %, as verified by an in vivo bioequivalence study [10]. In addition, analogous to the method used in the direct comparison, the chosen indirect comparison method is an average bioequivalence approach, which allows to compare the results of the indirect comparisons with the results of in vivo bioequivalence studies, because this is the criterion used for the studies for generic drug registration. Thus, this method is preferred over other methods recommended in literature based on population and individual bioequivalence approach [18, 19] and scaled average bioequivalence approach [20].

The indirect comparisons were only made between two generic formulations when their bioequivalence studies used the same dose, design, and conditions (i.e., either fasting/fed and/or either crossover/parallel). In line with recommendations by Gwaza et al.[21], a pragmatic method based on *t* test was used (see the algorithm below). This method has been shown to give comparable results as the homoscedastic method. All analyses were performed using Excel, Microsoft Office 2010®.

#### Algorithms

All calculations were based on *ln*-transformed data. The ratios between two generic formulations (G2/G1) for AUC (AUC_0-t_ for single dose studies and AUC_Ʈ_ for one dosing interval at steady state for multiple-dose studies) and C_max_ were calculated by subtraction of the *ln*-transformed generic/innovator ratios in one bioequivalence study (R_BE1_) from the ratios in another bioequivalence study (R_BE2_), which gives the adjusted difference between G1 and G2:

1 $$ \ln {R}_{\left(G2/G1\right)}= \ln {R}_{BE2}- \ln {R}_{BE1} $$

The 90 % CIs were calculated as follows:

2 $$ S{E}_{(d)}=\sqrt[2]{S{E}_1^2+S{E}_2^2}\kern1em ;\kern2em \left(d.f.={n}_1+{n}_2-2\right) $$

3 $$ \ln -\mathrm{transformed}\;90\%\;\mathrm{C}\mathrm{I}={ \ln \mathrm{R}}_{\left(G2/G1\right)}\pm t(df)\times S{E}_{(d)} $$

The standard errors from the ANOVA model in the bioequivalence studies (SE_1_ and SE_2_) were used to estimate the standard error (SE_(d)_) in the indirect comparison of G2 and G1. Degrees of freedom (*d.f.*) were estimated as the sum of subjects from the two bioequivalence studies (n_1_ and n_2_) minus two, and student’s *t* distribution t-percentiles *t(d.f.)* were used. The exponentiated results from Eqs. 1 and 3 were used to judge bioequivalence between generics.

#### Definition of bioequivalence

The European Medicines Agency (EMA) guidelines [2, 22] regarding bioequivalence were followed, i.e., 90 % CIs for both AUC and C_max_ of a generic drug with immediate release properties should meet the 80–125 % criterion. Further guidance on NTIDs was adhered for cyclosporine, tacrolimus, and mycophenolate mofetil [23]. The tightened acceptance range of 90–111 % was applied for both AUC and C_max_ for cyclosporine, and only AUC for tacrolimus. Bioequivalence of generic mycophenolate mofetil was demonstrated based on the plasma concentration of mycophenolic acid, and the 80–125 % criterion was followed.

## Results

Following the selection criteria, atorvastatin, bicalutamide, naratriptan, olanzapine, perindopril, and venlafaxine were included for this study. Further, the immunosuppressants cyclosporine, tacrolimus, and mycophenolate mofetil were included. Overall, eight of the selected APIs were immediate release formulations, whereas one, i.e., venlafaxine, was an extended release formulation.

### Clinical bioequivalence studies

For the selected 9 APIs, 115 brands of generic drugs were identified, which were registered based on 120 bioequivalence studies in total (Table 1). A number of different brands of generic products were registered based on the same dossier, i.e., the same bioequivalence study(ies). The generic/innovator ratios for AUC and C_max_ in the 120 studies ranged from 90.0 to 116.7 % and from 87.7 to 118.5 %, respectively. The mean absolute deviation of the ratios from 100 % in this set of generics was 4.5 % for AUC and 5.1 % for C_max_, respectively. The ranges of the lower and upper boundary of the 90 % CIs for AUC and C_max_ over the available bioequivalence studies are summarized for every API and strength in Table 1.

**Table 1:** A summary of selected generic drugs and bioequivalence studies (ranges of 90 % CIs for AUC and C_max_) in the study

APIs (dosage form)	Generic brands (*n* = 115)	Strengths (mg)	Generics (*n* = 354)	Year of studies	Study strength (mg)	BE studies (*n* = 120)	Design			90 % CI for AUC		90 % CI for C_max_	
										The range of LL (%)	The range of UL (%)	The range of LL (%)	The range of UL (%)
Atorvastatin (IR)	18	10	18	2006–2010	40	4	Crossover	Single-dose	Fasting	90.0–98.0	99.0–107.7	89.0–97.9	108.0–123.4
		20	18										
		30	2										
		40	18		80	7				94.5–112.2	106.3–121.5	88.3–104.6	107.1–123.6
		60	1										
		80	11										
Bicalutamide (IR)	18	50	18	2004–2010	50	6	Crossover	Single-dose	Fasting	84.5–100.5	96.0–112.5	83.4–99.6	92.2–108.7
						9	Parallel			85.8–100.3	102.5–122.8	90.0–99.1	104.0–113.9
		150	5		150*	1	Crossover			100.1^**†**^	120.0^**†**^	100.2^**†**^	111.1^**†**^
						1	Parallel			97.0^**†**^	124.1^**†**^	100.0^**†**^	121.7^**†**^
Ciclosporine (IR)	3	25	3	2001–2002	100	2	Crossover	Single-dose	Fasting	84.8–93.0	98.0–109.0	81.0–90.0	101.5–109.0
		50	3										
		100	3										
Mycophenolate mofetil (IR)	10	250	8	2005–2010	250	4	Crossover	Single-dose	Fasting	96.6–98.3	102.3–105.7	92.2–93.5	109.8–111.9
		500	10		500	6				92.2–101.9	100.3–108.3	87.7–94.2	106.0–116.5
Naratriptan (IR)	7	2.5	7	2004–2010	2.5	6	Crossover	Single-dose	Fasting	92.8–100.0	98.6–108.0	90.0–97.4	101.8–114.2
Olanzapine (IR)	20	2.5	19	2002–2010	5	6	Crossover	Single-dose	Fasting	86.1–97.7	97.5–106.5	84.2–96.7	96.6–106.6
		5	20										
		7.5	19		10	9				93.7–101.2	101.2–119.2	91.0–103.5	102.0–116.1
		10	20										
		15	16		15	3				94.7–99.0	103.6–110.3	88.4–91.6	99.3–117.0
		20	16										
Perindopril (IR)	12	2	12	2004–2009	2*	1	Crossover	Single-dose	Fasting	100.2^**†**^	106.8^**†**^	98.8^**†**^	112.8^**†**^
		4	12		4	4				90.7–99.7	98.1–109.6	88.0–97.0	100.0–110.9
		8	12		8	7				94.9–102.3	102.0–111.6	89.1–99.1	109.6–123.6
Tacrolimus (IR)	8	0.5	8	2007–2010	0.5	2	Crossover	Single-dose	Fasting	91.5–101.5	105.9–108.0	90.2–103.0	96.8–120.8
		1	8										
		5	8		5	3				93.1–99.2	103.6–110.9	91.7–110.6	111.5–121.0
Venlafaxine (ER)	19	37.5,	19	2002–2009	37.5	2	Crossover	Single-dose	Fasting	92.2–98.7	104.4–116.9	84.6–94.7	95.0–107.5
						2			Fed	95.2–98.1	110.0–107.2	92.3–99.1	107.6–112.3
		75,	19		75	4		Single-dose	Fasting	86.5–110.4	104.4–121.0	89.8–112.5	100.8–124.8
						4			Fed	92.3–103.5	103.5–116.0	87.1–101.4	98.1–112.2
		150	19			4		Multiple-dose	Fed	98.5–104.0	113.6–118.0	95.3–110.7	106.8–124.6
					150	7		Single-dose	Fasting	93.9–106.3	104.3–121.0	84.2–109.5	96.3–121.6
		225	2			9			Fed	87.8–107.7	101.1–119.0	82.7–108.3	97.9–123.3
						6		Multiple-dose	Fed	90.9–111.0	102.0–122.0	85.7–109.0	107.2–118.0
					225*	1		Single-dose	Fed	98.0^**†**^	118.9^**†**^	97.0^**†**^	116.4^**†**^

*APIs* active pharmaceutical ingredients, *IR* immediate release, *ER* extended release, *BE* bioequivalence, *CI* confidence interval, *LL* the lower limits of 90 % CI, *UL* the upper limits of 90 % CI

*Only one bioequivalence study is available with such study design. † Actual ratios and 90 % CIs are recorded

### Adjusted indirect comparisons between generic drugs

In total, 292 indirect comparisons between generic drugs were conducted based on 116 bioequivalence studies. Four bioequivalence studies could not be used as they were the only bioequivalence study for a specific strength of APIs (Table 2). In 80.5 % (235 out of 292) of the comparisons, the 90 % CIs for both AUC and C_max_ fell within the 80–125 % (or 90–111 %) acceptance range. In 90.1 % (263 out of 292) and in 87.0 % (254 out of 292) of the comparisons, the estimated 90 % CIs were within the predefined acceptance range for AUC and C_max_, respectively (Table 2).

**Table 2:** A summary of the results in cases where bioequivalence in generic-generic comparisons (90 % CIs for AUC and C_max_) was not demonstrated

APIs (dosage form)	Study strength (mg)	G-G comparisons (*n* = 292)	Study design			90 % CI for AUC				90 % CI for C_max_			
						Outside margin* (*n* = 29)	Ranges (%)			Outside margin* (*n* = 38)	Ranges (%)		
							LL	UL	Exceeding^‡^		LL	UL	Exceeding^‡^
Atorvastatin (IR)	40	6	Crossover	Single-dose	Fasting	0	–	–	–	1	77.0^**†**^	103.8^**†**^	3.0
	80	21				0	–	–	–	9	77.0–98.6	103.4–131.3	0.4–6.3
Bicalutamide (IR)	50	15	Crossover	Single-dose	Fasting	1	104.4^**†**^	127.5^**†**^	2.5	3	79.8–109.5	90.3–126.3	0.2–1.3
		36	Parallel			16	74.0–103.5	96.6–131.1	0.1–6.1	0	–	–	–
Cyclosporine (IR)	100	1	Crossover	Single-dose	Fasting	1 ^Δ^	99.7^**†**^	123.1^**†**^	12.1 ^Δ^	1 ^Δ^	94.5^**†**^	126.4^**†**^	15.4^Δ^
Mycophenolate mofetil (IR)	250	6	Crossover	Single-dose	Fasting	0	–	–	–	0	–	–	–
	500	15				0	–	–	–	2	79.4–79.8	106.1–110.2	0.2–0.6
Naratriptan (IR)	2.5	15	Crossover	Single-dose	Fasting	0	–	–	–	0	–	–	–
Olanzapine (IR)	5	15	Crossover	Single-dose	Fasting	0	–	–	–	0	–	–	–
	10	36				0	–	–	–	0	–	–	–
	15	3				0	–	–	–	1	79.4^**†**^	103.6^**†**^	0.6
Perindopril (IR)	4	6	Crossover	Single-dose	Fasting	0	–	–	–	0	–	–	–
	8	21				0	–	–	–	2			
Tacrolimus (IR)	0.5	1	Crossover	Single-dose	Fasting	1 ^Δ^	98.3^**†**^	115.0^**†**^	4.0^Δ^	1	76.8^**†**^	91.2^**†**^	3.2
	5	3				2 ^Δ^	86.6–89.1	101.6–101.8	0.9–3.4^Δ^	1	102.9^**†**^	127.2^**†**^	2.2
Venlafaxine (ER)	37.5	1	Crossover	Single-dose	Fasting	0	–	–	–	0	–	–	–
		1			Fed	0	–	–	–	0	–	–	–
	75	6		Single-dose	Fasting	3	79.2–100.2	91.6–134.1	0.8–9.1	2	74.4–76.8	86.7–92.8	3.2–5.6
		6			Fed	0	–	–	–	0	–	–	–
		6		Multiple-dose	Fed	0	–	–	–	1	79.3^**†**^	93.1^**†**^	0.7
	150	21		Single-dose	Fasting	0	–	–	–	4	71.8–110.4	84.9–130.3	2.8–7.2
		36			Fed	3	105.7–109.9	129.0–130.5	4.0–5.5	8	70.2–112.5	86.9–140.1	03.–8.8
		15		Multiple-dose	Fed	2	111.2–111.9	127.6–129.6	2.6–4.6	2	99.0–103.3	127.5–131.4	2.5–6.4

*APIs* active pharmaceutical ingredients, I*R* immediate release, *ER* extended release, *BE* bioequivalence, *G-G* generic and generic, *CI* confidence interval, *LL* the lower limits of 90 % CI, *UL* the upper limits of 90 % CI

*The margin is 80–125 %,^Δ^ for some cases 90–111 % margin is applied. † Actual ratios and 90 % CIs are recorded for single G-G comparisons. ‡The percentages of the maximum exceeding of the boundary are recorded, compared to the margin of 80–125 % (or 90–111 %)

#### Area under the plasma concentration-time curve (AUC)

**Fig. 1:**
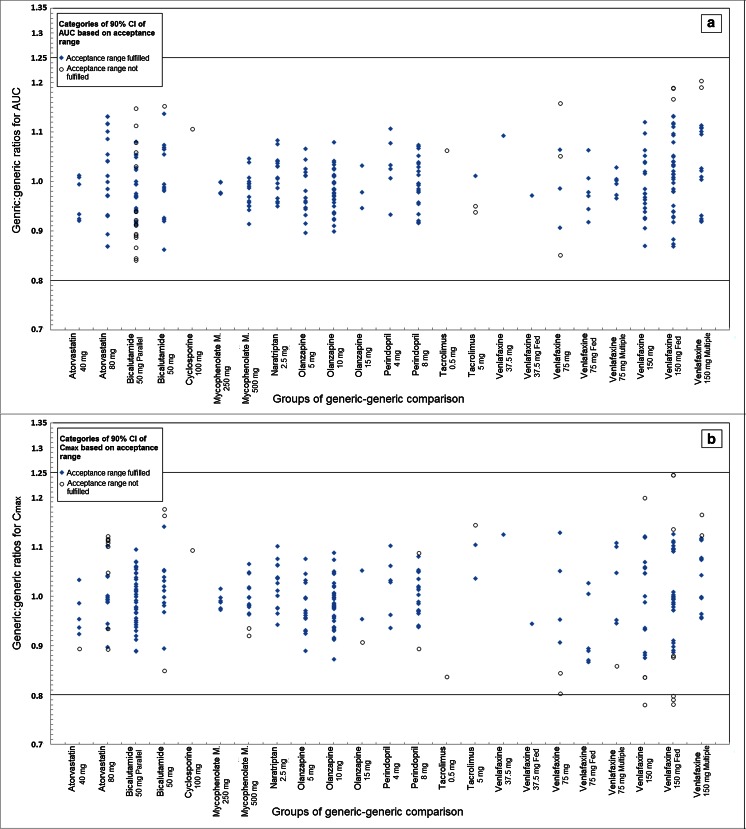
Calculated ratios of generic-generic drug comparisons for **a** AUC and **b** C_max_ (*n* = 292). *Dots in blue* represent the generic/generic ratios (*Y*-axis) at every comparison group (the same design with the same strength of APIs) (*X*-axis) for which the 90 % CIs were within the acceptance ranges; *circles in black* represent for the ratios of their 90 % CIs outside the margin. At the *X*-axis, the group is labeled by API, strength and the study design if not single dose, fasting or crossover

**Fig. 2:**
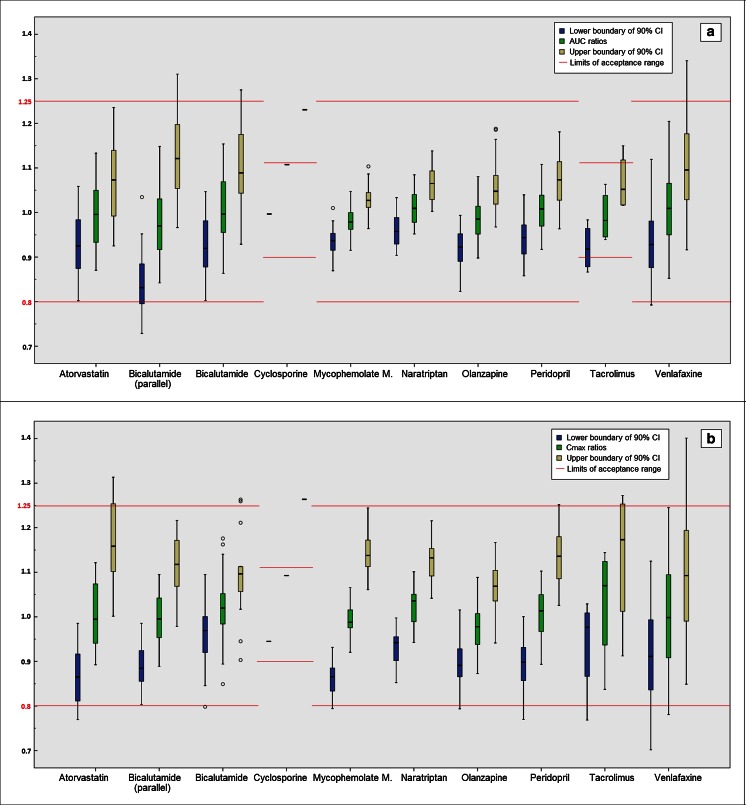
Boxplots of the calculated generic/generic ratios and 90 % CIs for **a** AUC and **b** C_max_ for each API (*n* = 292). The *boxplot in yellow and in blue* represents the distribution of upper and lower boundary of 90 % CIs for every API (*X*-axis), respectively. The *boxplot in green* represents the ratios. *Reference lines* (*red*) represent limits of the acceptance range required by the EMA

The estimated generic/generic ratios for AUC ranged from 84.2 to 120.4 % (Fig. 1a). The mean absolute deviation of the ratios from 100 % in this set of generic drugs was 5.4 %. The individual lower and upper boundaries of 90 % CIs for AUC varied in the ranges of 72.9–111.9 and 91.6–134.1 %, respectively (Fig. 2a).

All generic-generic 90 % CIs for AUC were within the 80–125 % acceptance criterion for atorvastatin, mycophenolate mofetil, naratriptan, olanzapine, and perindopril. For relatively few comparisons (29 out of 292, see Fig. 2a and Table 2), 90 % CIs for generic-generic AUC ratios did not meet the 80–125 % (or 90–111 %) criterion. The level by which the margin of 80 or 125 % (or 90 or 111 %) was exceeded ranged from 0.1 to 12.1 %. Twenty-six of these 29 cases did not exceed a wider range of 75–133 % (or 80–125 %). Thus, overall, in 1 % of the 292 indirect comparisons for AUC_t_, a wider range of 75–133 % (or 80–125 %) was exceeded.

#### Maximum plasma concentration (C_max_)

The generic/generic ratios for C_max_ ranged from 78.1 to 124.5 % (Fig. 1b) based on the indirect comparisons. The mean absolute deviation of the ratios from 100 % in this set of generic drugs was 6.1 %. The individual lower and upper boundaries of 90 % CIs for C_max_ ranged from 70.2 to 112.5 % and from 84.9 to 140.1 %, respectively. Similar with the situation for AUC, in the majority of generic-generic drug comparisons, a 90 % CI for C_max_ within the acceptance ranges was obtained (Fig. 2b and Table 2). A 90 % CI exceeding the 80–125 % margin (or 90–111 %) was observed for 38 of the 292 comparisons (Table 2). The level by which the margin of 80 or 125 % (or 90 or 111 %) was exceeded ranged from 0.1 to 15.4 %. Twenty-nine of these 38 cases did not exceed a wider range of 75–133 % (or 80–125 %). Thus, overall, in 3 % of the 292 indirect comparisons for C_max_, a wider range of 75–133 % (or 80–125 %) was exceeded.

## Discussion

Based on the presented results, bioequivalence between generic drugs can be concluded in 80.5 % of the cases. In 90.1 % of the comparisons, the 90 % CI of the ratios was within the 80–125 % (or 90–111 %) range for AUC and in 87.0 % for C_max_. Although bioequivalence was not formally demonstrated in 19.5 % of the cases with 90 % CIs outside the 80–125 % (or 90–111 %) margin (either AUC or C_max_), bio-inequivalence, potentially resulting in clinical consequences, was not demonstrated either. In this investigation, the adjusted indirect comparison combines the variability from both bioequivalence studies (Eq. 2). Therefore, the comparison results generally yield broader 90 % CIs for AUC and C_max_ between generic drugs, compared to the 90 % CIs obtained in actual clinical bioequivalence studies [9, 21]. According to Glenny et al. [24], the SE of the indirect estimate can be expected to be about 1.41 larger than that of the direct estimate. This suggests that when the 90 % CIs are obtained just outside the 80–125 % (or 90–111 %) margin (for instance within a wider range of 75–133 %, or 80–125 %), the actual 90 % CIs in a direct comparison situation of a bioequivalence study may well be within the acceptance range. Therefore, a failure of showing bioequivalence in our study does not demonstrate that the investigated generic drugs are not bioequivalent. In addition, the study results show that only 3 cases (1.0 %, *n* = 292) for AUC and 9 cases (3.1 %, *n* = 292) for C_max_ can be considered as extreme cases, i.e., exceeding the wider 75–133 % (or 80–125 %) criterion. Although our results cannot fully eliminate uncertainty regarding generic-generic interchangeability, since formal bioequivalence between generics was not demonstrated in all cases, no cases of bio-inequivalence were noted in our comparisons.

### Potential clinical relevance of observed differences for AUC and C_max_

Overall, the generic/generic ratios and 90 % CIs for C_max_ showed a larger variation than AUC in every API, and more estimations yielded 90 % CIs outside the acceptance criterion for C_max_. C_max_ is generally considered to be less critical than AUC for concluding therapeutic equivalence [23, 25]. In most cases, the requirement for C_max_ aims to regulate the safety profile of the generic formulations. It suggests that in most cases, clinical consequences are not expected when bioequivalence cannot be demonstrated for C_max_ with a relatively small magnitude of difference. For this reason, regulatory authorities (e.g., EMA and FDA) allow using a widened acceptance range for C_max_ for highly variable drugs [26, 25]. Specific API-related issues on the clinical relevance of 90 % CIs outside the 80–125 % margin (see Table 2) are discussed below.

#### Atorvastatin

For atorvastatin, deviations from the 80–125 % criterion were only seen for C_max_ (Table 2). Bio-inequivalence was not indicated in our comparisons. As discussed above, atorvastatin is a well-known highly variable drug (i.e., intrasubject variability >30 %), so a widened acceptance range (75–133 %) may be used [27]. Although the intrasubject variability is not known for the specific bioequivalence studies, this possibility suggests that exceeding of the 80–125 % may not be clinically relevant per se. Further, the magnitude of all exceeded cases was relatively small, i.e., within the range of 75–133 %, which supports that these cases are unlikely to be clinically relevant. In the literature, generic atorvastatin products have been shown to have comparable treatment outcomes with the innovator drug and were well-tolerated [28], and no significant differences in efficacy, adverse events, and patient management were seen upon clinical substitution [29, 30].

#### Bicalutamide

The elimination half-life of bicalutamide is very long (5–6 days) [31]. In patients, bicalutamide is given daily (50 mg tablet), after which an increase of the plasma concentration by 10-fold occurs as a consequence of the long half-life. As a result, the difference in C_max_ in single dose exposure between the generics is expected to be limited in actual clinical treatments. In addition, the 16 cases of 90 % CIs for AUC outside the 80–125 % margin appeared only in the comparisons of parallel studies. As the 90 % CIs are wider in a parallel design study due to intersubject variability, the likelihood of obtaining such 90 % CIs outside the margin is higher for this type of study. Further, despite this increased likelihood, the magnitude of all exceeded cases was relatively small, i.e., within the range of 75–133 %, which suggests that these cases may not be clinically relevant.

#### Cyclosporine

For cyclosporine, both generic formulations were registered based on the 80–125 % criterion, because they were registered before the EMA requirements on narrowed acceptance ranges for this drug [23] came into force. However, one of the two bioequivalence studies as support for registration of the cyclosporine generics actually fulfilled the 90–111 % range. It is obvious that the indirect comparison cannot meet the tightened acceptance range of 90–111 %.

The exceeded case is noted in Table 2, where the 90 % CI for AUC was within a wider range of 80–125 %, but the C_max_ (94.5–126.5 %) exceeded the margin. The adverse events of cyclosporine were screened in the database of The Netherlands Pharmacovigilance Centre Lareb, and no potential events related to formulation switching were found. However, it is acknowledged that pharmacovigilance databases like the one from Lareb suffer from under-reporting. Also, the fact that generic-generic exchange is not expected to occur regularly for this drug may add to the lack of reports regarding the issues for generic cyclosporine drugs. Thus, although bio-inequivalence is not indicated by our results, it is uncertain if the deviation from the 90 % acceptance criteria for cyclosporine leads to a different benefit-risk in the clinic.

#### Tacrolimus

Due to a long and variable elimination half-life of 11–16 h in the patients [32, 23], an accumulation of tacrolimus concentration would be expected in treatment and thus a difference in single dose C_max_ level is not expected to clinically impact the treatment. According to other literature [33–36], therapeutic equivalence between the innovator and generic tacrolimus has been observed without safety concerns. In clinical practice, dose titration is required for tacrolimus treatment in organ transplant patients. Therefore, therapeutic equivalence between different generic tacrolimus drugs is also likely to be the case under such dose titration, and clinical consequences due to our observed minor exceeding in AUC over the 90–111 % limits based on the estimation (Table 2) are not expected.

#### Venlafaxine

As venlafaxine is an extended release formulation, the pharmacological properties are more sensitive to limited differences in the generic drugs compared with immediate release formulation. This could lead to widespread 90 % CIs upon generic-generic drug substitution of venlafaxine. The exceeded cases are noted in Table 2, in which one case for AUC and seven cases for C_max_ exceeded a wider range of 75–133 %, which indeed indicates an uncertainty related to clinical consequences. However, for extended release formulations, the demonstration of bioequivalence under three conditions is required by EMA, i.e., single dose fasting and fed and steady state [2, 37, 22]. In our study, for most comparisons between generic drugs, bioequivalence under only one of the requested conditions could not be demonstrated. This situation is quite different from the immediate release formulation where only one pivotal bioequivalence study is requested. Since in all cases, bioequivalence is demonstrated under difference situations, the probability of inequivalence under only one condition affecting clinical outcomes is expected to be limited. Of note, FDA requires only bioequivalence under single-dose fed condition for the registration of venlafaxine generic drugs; thus, the situation of interchangeability for venlafaxine generic drugs in the USA can be different from Europe [38]. In literature, bio-inequivalence in fasted state for venlafaxine 75 mg has been reported between the generic and the innovator drug approved by FDA [39].

In this study, an initial cohort of APIs was defined based on the registration date of generic drugs, which was considered to be independent of bias, for instance potential difficulties of demonstration of bioequivalence. The initial cohort contained a large number of APIs (*n* = 21), allowing a valid selection from the cohort. The selected 6 APIs from the initial cohort are not considered to be the easy cases in terms of the demonstration of bioequivalence, for instance, atorvastatin, bicalutamide, and venlafaxine. A selection bias (except for the immunosuppressants that were included in the study) is not expected. Furthermore, the selected APIs had large numbers of generic drugs, e.g., atorvastatin (18 generic brands), bicalutamide (18), olanzapine (20), and venlafaxine (19) (Table 1). Although the selected APIs may not be the APIs with the largest number of generic drugs, they can still be considered to be representative to a general pattern of registered generic drugs.

The study results are in line with our previous study [9] and comparative bioavailability study [10] for investigation into the generic-generic interchangeability of topiramate and gabapentin. Furthermore, for tacrolimus, our findings are in line with a similar study performed by Herranz et al. [40]. Consistently, when considering all 120 bioequivalence studies in this investigation, the mean absolute deviation of the ratios from 100 % in this set of generics was 4.5 % for AUC and 5.1 % for C_max_. These figures are also comparable with the results of a study from the FDA [5], which showed that the mean absolute deviation from 100 % for AUC and C_max_ being 3.6 and 4.4 %, respectively. In our opinion, strengthened by justified unbiased selection of drugs, it is reasonable to assume that our estimation of generic-generic interchangeability from this study can be generalized as an overall pattern for a larger group of registered generic medicines.

Recently, a retrospective study reported a significant difference in serum level between generic phenytoin drugs and an increased seizure event rate following switching from one to other generic drug in Korean patients [41]. However, the possible selection bias was not justified for the study. The patients for whom the records of serum phenytoin levels were available may have been more susceptible for instable treatment effects, resulting in higher risk of seizures. Furthermore, the causal relationship between the generic phenytoin switching and decrease in serum level (and increase in seizure events) cannot be concluded, since it is unclear when the serum phenytoin levels were recorded, what daily dose was administrated and which generic drug was used in the pre- and post-interchange period in every patient. Thus, further research is warranted for the generic drugs involved in that study, and the conclusion may not be extrapolated to other generic drugs. In some papers, it is proposed that for registration, generic drugs should not only be compared to their innovator, but also to other generics that are already on the market. In our opinion, such a request would not be realistic. Further, this would even need to be repeated when another generic is applied for. Based on the outcome of this study, the actual chance of having a 90 % CI outside the criteria upon exchange of generics in direct comparisons is expected to be small. However, the possibility of exceeding the 80–125 % margin in the real life conditions cannot be excluded. Of note, the 80–125 % criterion is not directly linked to efficacy or safety. Thus, exceedance of the 80–125 % margin cannot be directly interpreted as resulting in clinical consequences per se.

## Conclusion

Based on a conservative approach, our study demonstrates that more than 80 % of the registered generic drugs were not only bioequivalent to the innovator but also to each other. Due to methodological constraints in our comparison, the 90 % CIs obtained in this study are generally larger than in the actual within-study comparisons. Therefore, we expect that the actual percentage of generics being bioequivalent to other generics in the actual clinical setting will be higher than 80 %. However, our results also imply that formal bioequivalence between generics could not be demonstrated in maximally 20 % of the cases. Still, the magnitude by which the 90 % CIs boundary is exceeded should not be interpreted as the actual difference between generic drugs in clinical practice [21], and in the vast majority of the cases, the exceedance of the acceptance criteria is limited. Further, although bioequivalence could not be demonstrated in some cases, in none of the cases the reverse, i.e., bio-inequivalence was demonstrated. Thus, although the results are not fully reassuring, we consider a pronounced risk upon generic-generic exchange in clinical practice as unlikely. Overall, our study suggests that exposure-related risks associated with the exchange of different generic drugs in clinical practice is limited, and not much increased—if any—to the situation in which a generic is exchanged with the innovator.

## Electronic supplementary material

ESM 1 (DOCX 48 kb)

## References

[1] Guidance for Industry (2003) Bioavailability and Bioequivalence. Studies for Orally Adminsitered Drug Products-General Considerations. US Department of Health and Human Services, Food and Drug Administration Center for Drug Evaluation and Research (CDER)

[2] Guidline on the Investigation of Bioequivalence (2010) London: committee for medicinal products for human use (CHMP), European Medicine Agency

[3] Annex7 (2006) Multisource (generic) pharmaceutical products: guidelines on registration requirements to establish interchangeability. Geneva: World Health Organization

[4] Guidance Document-Comparative Bioavailability Standards: Formulations Used for Systemic Effects Canada: Health Canada (2012)

[5] Davit BM, Nwakama PE, Buehler GJ, Conner DP, Haidar SH, Patel DT (2009). Comparing generic and innovator drugs: a review of 12 years of bioequivalence data from the United States Food and Drug Administration. Ann Pharmacother.

[6] Anderson S, Hauck WW (1996). The transitivity of bioequivalence testing: potential for drift. Int J Clin Pharmacol Ther.

[7] Karalis V, Macheras P, Bialer M (2014). Generic products of antiepileptic drugs: a perspective on bioequivalence, bioavailability, and formulation switches using Monte Carlo simulations. CNS Drugs.

[8] Karalis V, Bialer M, Macheras P (2013). Quantitative assessment of the switchability of generic products. Eur J Pharm Sci : Off J Eur Fed Pharm Sci.

[9] Maliepaard M, Banishki N, Gispen-de Wied CC, Teerenstra S, Elferink AJ (2011). Interchangeability of generic anti-epileptic drugs: a quantitative analysis of topiramate and gabapentin. Eur J Clin Pharmacol.

[10] Yu Y, Teerenstra S, Vanmolkot F, Neef C, Burger D, Maliepaard M (2013). Interchangeability of gabapentin generic formulations in the Netherlands: a comparative bioavailability study. Clin Pharmacol Ther.

[11] Harrison JJ, Schiff JR, Coursol CJ, Daley CJ, Dipchand AI, Heywood NM (2012). Generic immunosuppression in solid organ transplantation: a Canadian perspective. Transplantation.

[12] Kovarik JM, Noe A, Wang Y, Mueller I, DeNucci G, Schmouder RL (2006). Differentiation of innovator versus generic cyclosporine via a drug interaction on sirolimus. Eur J Clin Pharmacol.

[13] Hulbert AL, Pilch NA, Taber DJ, Chavin KD, Baliga PK (2012). Generic immunosuppression: deciphering the message our patients are receiving. Ann Pharmacother.

[14] Cutler C, Kesselheim A, Gabardi S, Andersson BS, Carpenter P, Khoury HJ (2011). Generic immunosuppressants in hematopoietic cell transplantation. Biol Blood Marrow Transplant : J Am Soc Blood Marrow Transplant.

[15] Christians U (1999). Generic immunosuppressants: the European perspective. Transplant Proc.

[16] Christians U, Klawitter J, Clavijo CF (2010). Bioequivalence testing of immunosuppressants: concepts and misconceptions. Kidney Int Suppl.

[17] Robertsen I, Asberg A, Ingero AO, Vethe NT, Bremer S, Bergan S (2014). Use of generic tacrolimus in elderly renal transplant recipients: precaution is needed. Transplantation.

[18] Dong X, Tsong Y, Shen M (2014). Equivalence tests for interchangeability based on two one-sided probabilities. J Biopharm Stat.

[19] Dong X, Tsong Y (2014). Equivalence assessment for interchangeability based on two-sided tests. J Biopharm Stat.

[20] Bialer M, Midha KK (2010). Generic products of antiepileptic drugs: a perspective on bioequivalence and interchangeability. Epilepsia.

[21] Gwaza L, Gordon J, Welink J, Potthast H, Hansson H, Stahl M (2012). Statistical approaches to indirectly compare bioequivalence between generics: a comparison of methodologies employing artemether/lumefantrine 20/120 mg tablets as prequalified by WHO. Eur J Clin Pharmacol.

[22] Note for guidance on modified release oral and trandermal doseage forms: section II (pharmacokinetic and clinical evaluation) CPMP/EWP/280/96 Corr * (1999) London: Committee for proprietary medicinal products (CPMP), European Medicine Agency

[23] Question & Answers: Positions on specific questions addressed to the pharmacokinetics working party (2014) London: Committee for Medicinal Products for Human use (CHMP), European Medicine Agency

[24] Glenny AM, Altman DG, Song F, Sakarovitch C, Deeks JJ, D’Amico R (2005). Indirect comparisons of competing interventions. Health Technol Assess.

[25] Garcia-Arieta A, Gordon J (2012). Bioequivalence requirements in the European Union: critical discussion. AAPS J.

[26] Haidar SH, Davit B, Chen ML, Conner D, Lee L, Li QH (2008). Bioequivalence approaches for highly variable drugs and drug products. Pharm Res.

[27] Draft Guidance on Atorvastatin Calcium (2010) U.S. Food and Drug Administration

[28] Kim SH, Park K, Hong SJ, Cho YS, Sung JD, Moon GW (2010). Efficacy and tolerability of a generic and a branded formulation of atorvastatin 20 mg/d in hypercholesterolemic Korean adults at high risk for cardiovascular disease: a multicenter, prospective, randomized, double-blind, double-dummy clinical trial. Clin Ther.

[29] Jackevicius CA, Tu JV, Krumholz HM (2013). Statins: is it safe and effective to use generic “equivalents”?. Can J Cardiol.

[30] Rahalkar AR, Ban MR, Hegele RA (2013). Clinical equivalence of proprietary and generic atorvastatin in lipid clinic patients. Can J Cardiol.

[31] Summary of Product Characteristics: Bicalutamide 50mg film-coated tablets (2013) http://www.medicines.org.uk/emc/medicine/22272/spc#PHARMACOKINETIC_PROPS

[32] Summary of Product Characteristics: Prograf 0.5mg, 1mg, 5mg Hard Capsules (2013) http://www.medicines.org.uk/emc/medicine/11102/SPC/Prograf+0.5mg%2c+1mg%2c+5mg+Hard+Capsules/

[33] McDevitt-Potter LM, Sadaka B, Tichy EM, Rogers CC, Gabardi S (2011). A multicenter experience with generic tacrolimus conversion. Transplantation.

[34] Staffan Rosenborg ANM, Tora A, Lars W, Peter B (2014). Systematic conversion to generic tacrolimus in stable kidney transplant recipients. Clin Kidney J.

[35] Heavner MS, Tichy EM, Yazdi M, Formica RN, Kulkarni S, Emre S (2013). Clinical outcomes associated with conversion from brand-name to generic tacrolimus in hospitalized kidney transplant recipients. Am J Health Syst Pharm.

[36] Spence MM, Nguyen LM, Hui RL, Chan J (2012). Evaluation of clinical and safety outcomes associated with conversion from brand-name to generic tacrolimus in transplant recipients enrolled in an integrated health care system. Pharmacotherapy.

[37] Guideline on the pharmacokinetic and clinical evaluation of modified release dosage forms (EMA/CPMP/EWP/280/96 Corr1) Draft XXIII (2013) London: committee for medicinal products for human use (CHMP), European Medicine Agency

[38] Draft Guidance on Venlafaxine Hydrochloride (2010) U.S. Food and Drug Administration

[39] Wright CW, Aikman MS, Werts E, Seabolt J, Haeusler JM (2009). Bioequivalence of single and multiple doses of venlafaxine extended-release tablets and capsules in the fasted and fed states: four open-label, randomized crossover trials in healthy volunteers. Clin Ther.

[40] Herranz M, Morales-Alcelay S, Corredera-Hernandez MT, de la Torre-Alvarado JM, Blazquez-Perez A, Suarez-Gea ML (2013). Bioequivalence between generic tacrolimus products marketed in Spain by adjusted indirect comparison. Eur J Clin Pharmacol.

[41] Shin JW, Chu K, Jung KH, Lee ST, Moon J, Lee SK (2014). Switching between phenytoin generics in patients with epilepsy may lead to increased risk of breakthrough seizure: chart analysis and practice recommendations. Int J Clin Pharmacol Ther.

