# Supplementary material for: Investigation into the interchangeability of generic formulations using immunosuppressants and a broad selection of medicines
Source: Eur J Clin Pharmacol. 2015 Jun 12;71(8):979–90. doi: 10.1007/s00228-015-1878-z (PMC4500859; doi:10.1007/s00228-015-1878-z)
Supplement: Supplementary file 1 — (DOCX 48 kb) [file 228_2015_1878_MOESM1_ESM.docx]

**Supplementary Material 1.** The data of the bioequivalence studies for the selected medicines in the investigation.

|  |  |  |  | **90% CI for AUC** | | **90% CI for Cmax** | |
| --- | --- | --- | --- | --- | --- | --- | --- |
| **Product name** | **G code** | **Doses (mg)** | **Number of subjects** | **Lower limit (%)** | **Upper limit (%)** | **Lower limit (%)** | **Upper limit (%)** |
| **Atorvastatine** | AG1 | 40 | 90 | 97.3 | 103.2 | 95.5 | 118.1 |
|  | AG2 | 40 | 57 | 97.3 | 107.7 | 97.9 | 123.4 |
|  | AG3 | 40 | 81 | 98.0 | 106.0 | 95.0 | 116.0 |
|  | AG4 | 40 | 63 | 90.0 | 99.0 | 89.0 | 108.0 |
|  | AG5 | 80 | 48 | 96.0 | 114.0 | 89.0 | 115.0 |
|  | AG6 | 80 | 112 | 112.2 | 121.5 | 104.6 | 121.0 |
|  | AG7 | 80 | 92 | 102.7 | 112.7 | 88.9 | 107.1 |
|  | AG8 | 80 | 55 | 94.5 | 109.0 | 88.3 | 114.3 |
|  | AG9 | 80 | 93 | 108.7 | 118.6 | 102.7 | 123.6 |
|  | AG10 | 80 | 68 | 97.0 | 106.3 | 101.7 | 121.8 |
|  | AG11 | 80 | 54 | 109.4 | 121.0 | 92.2 | 120.0 |
| **Bicalutamide** | BG1 | 50 | 24 | 92.7 | 101.6 | 95.7 | 100.5 |
|  | BG2 | 50 | 42 | 100.5 | 108.2 | 99.6 | 107.1 |
|  | BG3 | 50 | 39 | 84.5 | 96.0 | 83.4 | 92.2 |
|  | BG4 | 50 | 25 | 96.0 | 112.5 | 95.6 | 108.7 |
|  | BG5 | 50 | 30 | 88.0 | 106.0 | 96.5 | 104.6 |
|  | BG6 | 50 | 49 | 96.9 | 108.4 | 97.9 | 108.7 |
|  | BG7 | 50 | T=30, R=30 | 100.3 | 122.8 | 93.1 | 110.4 |
|  | BG8 | 50 | T=24,R=24 | 85.8 | 102.5 | 91.9 | 107.1 |
|  | BG9 | 50 | T=23; R=24 | 97.6 | 119.7 | 92.6 | 108.9 |
|  | BG10 | 50 | T=24;R=25 | 86.9 | 117.9 | 99.1 | 113.9 |
|  | BG11 | 50 | T=23, R=24 | 92.0 | 107.0 | 90.0 | 104.0 |
|  | BG12 | 50 | T=24, R=23 | 87.3 | 111.2 | 91.0 | 111.4 |
|  | BG13 | 50 | R=24, T=24 | 92.0 | 107.0 | 90.0 | 104.0 |
|  | BG14 | 50 | R=22, T=22 | 87.3 | 111.2 | 91.0 | 111.4 |
|  | BG15 | 50 | R=34, T=34 | 91.6 | 113.5 | 92.9 | 109.3 |
|  | Excluded* | 150 | T=33, R=33 | 97.0 | 124.1 | 100.0 | 121.7 |
|  | Excluded* | 150 | 21 | 100.1 | 120.0 | 100.2 | 111.1 |
| **Cyclosporine** | CG1 | 100 | 24 | 84.8 | 98.0 | 81.0 | 101.5 |
|  | CG2 | 100 | 24 | 93.0 | 109.0 | 90.0 | 109.0 |
| **Mycophenolate mofetil**  **(mycophenolic acid)** | MG1 | 250 | 33 | 97.6 | 105.7 | 93.0 | 111.9 |
|  | MG2 | 250 | 50 | 98.0 | 105.2 | 93.5 | 110.8 |
|  | MG3 | 250 | 67 | 98.3 | 103.2 | 92.5 | 109.8 |
|  | MG4 | 250 | 36 | 96.6 | 102.3 | 92.2 | 110.1 |
|  | MG5 | 500 | 116 | 98.3 | 102.4 | 94.1 | 116.5 |
|  | MG6 | 500 | 37 | 101.9 | 108.3 | 89.9 | 107.4 |
|  | MG7 | 500 | 82 | 97.2 | 102.7 | 87.7 | 106.0 |
|  | MG8 | 500 | 39 | 95.7 | 102.6 | 91.9 | 111.1 |
|  | MG9 | 500 | 57 | 92.2 | 100.3 | 94.2 | 107.9 |
|  | MG10 | 500 | 33 | 96.0 | 104.0 | 91.0 | 116.0 |
| **Naratriptan** | NG1 | 2.5 | 28 | 92.8 | 98.6 | 90.0 | 101.8 |
|  | NG2 | 2.5 | 35 | 96.4 | 103.5 | 92.8 | 106.1 |
|  | NG3 | 2.5 | 25 | 99.6 | 106.6 | 97.4 | 114.2 |
|  | NG4 | 2.5 | 26 | 100.0 | 108.0 | 95.0 | 109.0 |
|  | NG5 | 2.5 | 30 | 95.3 | 102.4 | 93.2 | 105.9 |
|  | NG6 | 2.5 | 26 | 95.6 | 103.9 | 95.7 | 110.9 |
| **Olanzapine** | OG1 | 5 | 24 | 86.4 | 105.6 | 89.1 | 106.0 |
|  | OG2 | 5 | 24 | 95.6 | 104.4 | 90.5 | 99.2 |
|  | OG3 | 5 | 24 | 97.7 | 106.5 | 96.7 | 106.6 |
|  | OG4 | 5 | 39 | 92.1 | 101.7 | 88.4 | 99.7 |
|  | OG5 | 5 | 40 | 92.4 | 104.3 | 89.5 | 105.0 |
|  | OG6 | 5 | 30 | 86.1 | 97.5 | 84.2 | 96.9 |
|  | OG7 | 10 | 22 | 97.4 | 104.8 | 98.2 | 110.9 |
|  | OG8 | 10 | 24 | 101.2 | 108.2 | 98.6 | 111.1 |
|  | OG9 | 10 | 20 | 100.0 | 119.2 | 103.5 | 116.1 |
|  | OG10 | 10 | 22 | 99.4 | 105.7 | 96.4 | 108.2 |
|  | OG11 | 10 | 22 | 95.5 | 101.2 | 94.7 | 109.8 |
|  | OG12 | 10 | 16 | 98.0 | 105.0 | 91.0 | 102.0 |
|  | OG13 | 10 | 36 | 96.2 | 103.0 | 99.2 | 109.2 |
|  | OG14 | 10 | 35 | 98.6 | 106.1 | 94.0 | 106.4 |
|  | OG15 | 10 | 23 | 93.7 | 108.6 | 96.9 | 108.8 |
|  | OG16 | 15 | 15 | 99.0 | 110.0 | 91.0 | 117.0 |
|  | OG17 | 15 | 17 | 94.7 | 103.6 | 88.4 | 99.3 |
|  | OG18 | 15 | 22 | 94.9 | 110.3 | 91.6 | 106.1 |
| **Perindopril tert-butylamine** | Excluded* | 2 | 35 | 100.2 | 106.8 | 98.8 | 112.8 |
|  | PG1 | 4 | 42 | 96.7 | 105.4 | 90.5 | 105.5 |
|  | PG2 | 4 | 35 | 90.7 | 98.1 | 94.1 | 107.3 |
|  | PG3 | 4 | 35 | 98.0 | 106.0 | 88.0 | 100.0 |
|  | PG4 | 4 | 26 | 99.7 | 109.6 | 97.0 | 110.9 |
|  | PG5 | 8 | 34 | 94.9 | 102.0 | 97.2 | 114.0 |
|  | PG6 | 8 | 40 | 102.3 | 108.7 | 92.9 | 111.4 |
|  | PG7 | 8 | 30 | 96.5 | 106.0 | 95.5 | 116.2 |
|  | PG8 | 8 | 25 | 99.2 | 111.6 | 99.0 | 123.6 |
|  | PG9 | 8 | 29 | 97.7 | 109.4 | 89.1 | 109.6 |
|  | PG10 | 8 | 34 | 100.0 | 110.0 | 99.1 | 115.0 |
|  | PG11 | 8 | 28 | 98.2 | 106.2 | 97.4 | 110.6 |
| **Tacroliums** | TG1 | 0.5 | 36 | 91.5 | 105.9 | 103.0 | 120.8 |
|  | TG2 | 0.5 | 207 | 101.5 | 108.0 | 90.2 | 96.8 |
|  | TG3 | 5 | 42 | 99.2 | 110.9 | 91.7 | 111.5 |
|  | TG4 | 5 | 141 | 93.1 | 104.7 | 105.6 | 117.9 |
|  | TG5 | 5 | 109 | 96.2 | 103.6 | 110.6 | 121.0 |
| **Venlafaxine** | VG1 | 37.5 | 32 | 92.2 | 104.4 | 84.6 | 95.0 |
|  | VG2 | 37.5 | 34 | 98.7 | 116.9 | 94.7 | 107.5 |
|  | VG3 | 75 | 24 | 86.5 | 115.0 | 97.0 | 113.5 |
|  | VG4 | 75 | 38 | 110.4 | 121.0 | 112.5 | 124.8 |
|  | VG5 | 75 | 35 | 93.0 | 104.4 | 89.8 | 100.8 |
|  | VG6 | 75 | 38 | 94.0 | 118.0 | 92.0 | 108.0 |
|  | VG7 | 150 | 36 | 106.3 | 119.4 | 109.5 | 121.6 |
|  | VG8 | 150 | 36 | 100.7 | 113.3 | 89.8 | 103.7 |
|  | VG9 | 150 | 24 | 93.9 | 104.3 | 90.8 | 102.6 |
|  | VG10 | 150 | 42 | 102.3 | 115.5 | 96.0 | 108.8 |
|  | VG11 | 150 | 36 | 98.3 | 113.0 | 84.2 | 96.3 |
|  | VG12 | 150 | 39 | 101.0 | 121.0 | 96.0 | 107.0 |
|  | VG13 | 150 | 37 | 98.2 | 109.0 | 103.0 | 114.0 |
|  | G1 | 37.5 | 36 | 98.1 | 110.0 | 99.1 | 112.3 |
|  | G2 | 37.5 | 43 | 95.2 | 107.2 | 92.3 | 107.6 |
|  | G3 | 75 | 24 | 103.5 | 111.0 | 101.4 | 111.3 |
|  | G4 | 75 | 38 | 99.5 | 109.1 | 101.2 | 112.2 |
|  | G5 | 75 | 38 | 92.3 | 103.5 | 87.1 | 98.1 |
|  | G6 | 75 | 40 | 97.0 | 116.0 | 90.0 | 102.0 |
|  | G7 | 150 | 35 | 99.8 | 113.1 | 95.8 | 108.8 |
|  | G8 | 150 | 36 | 103.1 | 112.4 | 107.6 | 123.3 |
|  | G9 | 150 | 21 | 95.0 | 105.8 | 106.7 | 119.1 |
|  | G10 | 150 | 23 | 94.5 | 103.5 | 98.1 | 107.4 |
|  | G11 | 150 | 35 | 103.9 | 112.9 | 108.3 | 119.9 |
|  | G12 | 150 | 43 | 87.8 | 101.1 | 82.7 | 97.9 |
|  | G13 | 150 | 66 | 107.7 | 116.8 | 96.0 | 108.8 |
|  | G14 | 150 | 46 | 105.0 | 119.0 | 106.0 | 119.0 |
|  | G15 | 150 | 41 | 102.0 | 118.0 | 103.0 | 122.0 |
|  | VssG1 | 75 | 24 | 103.9 | 113.6 | 100.8 | 111.3 |
|  | VssG2 | 75 | 22 | 104.0 | 114.9 | 110.7 | 124.6 |
|  | VssG3 | 75 | 34 | 98.5 | 113.7 | 95.3 | 106.8 |
|  | VssG5 | 150 | 28 | 97.4 | 111.2 | 94.4 | 107.2 |
|  | VssG6 | 150 | 36 | 98.8 | 110.7 | 93.4 | 107.8 |
|  | VssG7 | 150 | 34 | 90.9 | 102.0 | 85.7 | 107.9 |
|  | VssG8 | 150 | 38 | 110.3 | 119.4 | 91.6 | 109.7 |
|  | VssG4 | 75 | 30 | 101.0 | 118.0 | 105.0 | 118.0 |
|  | VssG9 | 150 | 35 | 111.0 | 122.0 | 109.0 | 118.0 |
|  | VssG10 | 150 | 39 | 102.0 | 113.0 | 102.0 | 114.0 |
|  | Excluded* | 225 | 34 | 98.0 | 118.9 | 97.0 | 116.4 |

* Four studies were excluded from the adjusted indirect comparisons.
